# Imaging of Biological Cells Using Luminescent Silver Nanoparticles

**DOI:** 10.1186/s11671-016-1243-x

**Published:** 2016-01-19

**Authors:** Vira Kravets, Zamavang Almemar, Ke Jiang, Kyle Culhane, Rosa Machado, Guy Hagen, Andriy Kotko, Igor Dmytruk, Kathrin Spendier, Anatoliy Pinchuk

**Affiliations:** Physics Department, University of Colorado, 1420, Austin Bluffs Parkway, 80918 Colorado Springs, CO USA; Center for Biofrontiers Institute, University of Colorado, 1420, Austin Bluffs Parkway, 80918 Colorado Springs, CO USA; Mechanical and Aerospace Engineering Department, University of Colorado, 1420, Austin Bluffs Parkway, 80918 Colorado Springs, CO USA; Taras Shevchenko National University of Kyiv, 4 Academic Glushkov Prospect, Kyiv, 01601 Ukraine; I.M. Frantsevich Institute for Problems of Material Science, 3 Krzhizhanovsky str., 03680 Kyiv, Ukraine

**Keywords:** Photoluminescence, Surface plasmon resonance, Plasmon radiative decay, Surface-enhanced photoluminescence, Nanoparticles, Bio-imaging, Neural stem cells, Rat basophilic leukemia cells, 78.67.Bf, 71.45.-d

## Abstract

The application of luminescent silver nanoparticles as imaging agents for neural stem and rat basophilic leukemia cells was demonstrated. The experimental size dependence of the extinction and emission spectra for silver nanoparticles were also studied. The nanoparticles were functionalized with fluorescent glycine dimers. Spectral position of the resonance extinction and photoluminescence emission for particles with average diameters ranging from 9 to 32 nm were examined. As the particle size increased, the spectral peaks for both extinction and the intrinsic emission of silver nanoparticles shifted to the red end of the spectrum. The intrinsic photoluminescence of the particles was orders of magnitude weaker and was spectrally separated from the photoluminescence of the glycine dimer ligands. The spectral position of the ligand emission was independent of the particle size; however, the quantum yield of the nanoparticle-ligand system was size-dependent. This was attributed to the enhancement of the ligand’s emission caused by the local electric field strength’s dependence on the particle size. The maximum quantum yield determined for the nanoparticle-ligand complex was (5.2 ± 0.1) %. The nanoparticles were able to penetrate cell membranes of rat basophilic leukemia and neural stem cells fixed with paraformaldehyde. Additionally, toxicity studies were performed. It was found that towards rat basophilic leukemia cells, luminescent silver nanoparticles had a toxic effect in the silver atom concentration range of 10–100 μM.

## Background

Noble metal nanoparticles (NPs) exhibit unique electronic and optical properties when excited by external light because of the collective and coherent excitations of their conduction electrons known as surface plasmon resonance (SPR) [1, 2]. SPR excitations in metal NPs can find applications in biosensing, [3, 4] bio-imaging, [5] photothermal therapy, [6], and in plasmonic rulers, [7, 8] to name just a few. Silver NPs exhibit the strongest SPR response when compared to other noble metals, such as gold or copper. The size of the metal NPs plays a crucial role in their photoluminescence emission due to the strong size dependence of the local field enhancement factor [9–12]. The size dependence of SPR in noble metal NPs is a well-known phenomenon; [13] however, the size-dependent photoluminescence (PL) has been studied much less, despite its potential applications in solar cells, [2] SERS, [4], and bio-imaging [5, 14].

In the work presented here, the SPR in silver NPs was exploited to control their intrinsic PL emission, as well as to enhance the PL of glycine ligands. In previous studies, the intrinsic PL of NPs has been rationalized as SPR-enhanced interband electronic transitions, [15] or alternatively direct plasmon emission due to the radiative damping of the SPR [11]. Previous reports have indicated a quantum yield (QY) for the intrinsic PL of silver NPs as high as 1.2 × 10^−2^ for 8 nm NPs, and 4.6 × 10^−3^ for 11 nm NPs, [15] a somewhat lower quantum yield 10^−4^–10^−5^ for 5 nm gold NPs, [16], and even lower 10^−5^ for 17 nm copper NPs [17]. All of the mentioned QY values are orders of magnitude larger than that of 10^−10^ for bulk metals [18].

Another SPR-related effect, the enhancement of a ligand’s PL from a nanoparticle’s surface is caused by the strong local field at the NPs surface, whereby the particle enhances the incoming and outgoing photons. The surface enhancement of Raman scattering for Rhodamine 6G was reported to be most efficient for 50-nm-diameter NP [19]. In the experiments presented here, evidence of the connection between SPR excitations in silver NPs and their intrinsic PL signal was found. This connection was then compared to the surface-enhanced PL of the glycine dimer ligands, with the goal of developing robust, non-toxic, stable, and bright fluorescent biomarkers.

## Methods

### Materials

All experiments were performed with chemicals and solvents of reagent grade and were used without further modification or purification. For synthetic procedures, glycine (CAS: 56-40-6, Reagent Plus 99 %) and silver nitrate (CAS: 7761-88-8, ACS Reagent 99+ %) were purchased from Sigma-Aldrich. The quantum yield standard, quinine sulfate (CAS: 207671-44-1, meets USP testing specifications), was purchased from Sigma-Aldrich. Aqueous quinine standards were prepared in 0.05 M sulfuric acid (H2SO4_(aq)_, CAS: 7664-93-9, Alfa Aesar). Distilled deionized water (pH 6.3) was used for all experiments and reagent dilutions.

### NP’s Synthesis

The nanoparticles used for this study were synthesized based on a procedure previously described in the literature [20]. Silver nitrate and glycine (1-to-10 mass ratio) were dissolved in distilled deionized water in order to form an intimate mixture. The water was then evaporated under low pressure to form a solid crystalline reaction mixture. After solvent evaporation, a solid phase thermal reduction was performed on the crystalline mixture at 445 K for 5 min. During this procedure, glycine-dimer-coated silver nanoparticles were formed via a reduction-oxidation reaction, while the solid glycine matrix controlled the size of these particles. The resulting product was dissolved in water and sonicated for 24 h to break apart any aggregates. The absorption and emission spectra, obtained every hour during the sonication procedure, showed no evidence of additional product formation (such as nanoclusters). The product was then filtered through a 2-μm-core-diameter filter and subjected to a centrifugation procedure that separated the insoluble aggregates and free glycine molecules from the glycine-dimer-coated nanoparticles.

### Cell Culture

The C17.2 cell line adopted from the cerebellum of newborn mice was supplied by Dr. Evan Y. Snyder from Harvard Medical School, Boston, MA. C17.2 neural stem cells were grown in Dulbecco’s modified Eagle’s medium (DMEM) and supplemented with HEPES, penicillin streptomycin (Pen-Strep), l-glutamine, 10 % fetal bovine serum (FBS), and 5 % normal horse serum.

Rat basophilic leukemia (RBL) cells (RBL-2H3; ATCC number CRL-2256) were maintained in minimal essential medium (MEM) supplemented with 10 % FBS, 1 % Pen-Strep, and 1 % l-glutamine (L-glut).

### Centrifugation Procedure

**Fig. 1:**
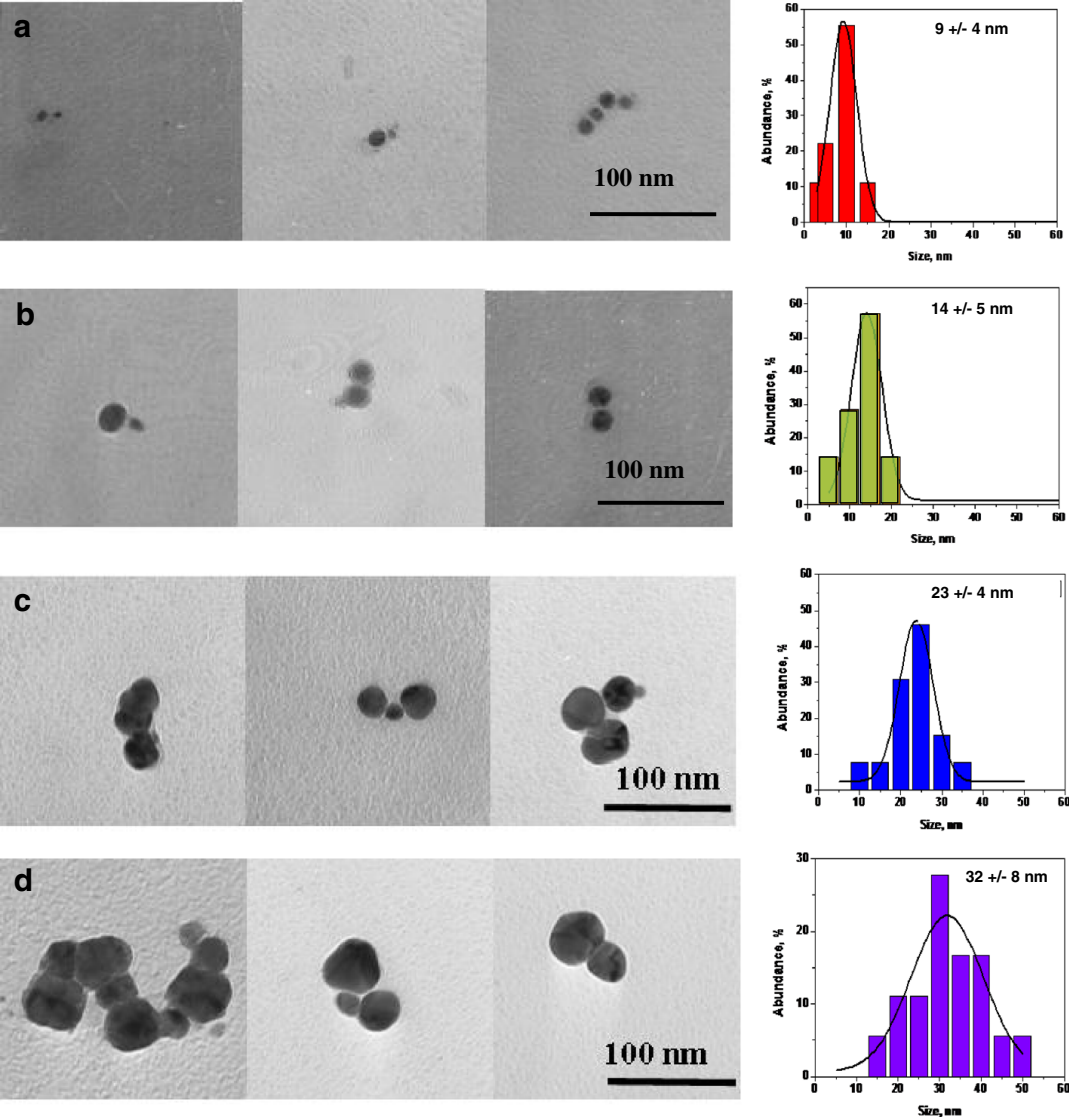
Representative TEM images of silver nanoparticles of different sizes (**a**-**d**) along with their size distributions, obtained from the TEM data

**Fig. 2:**
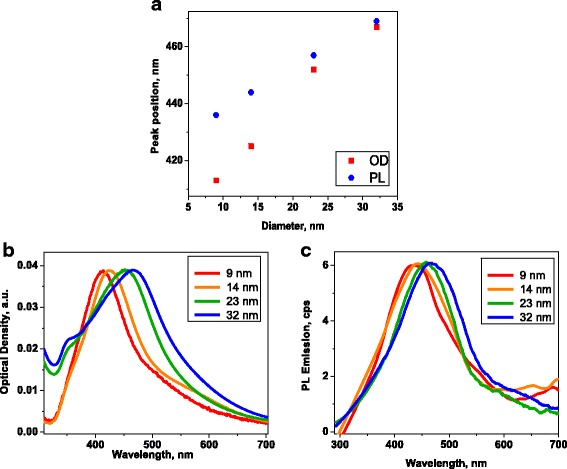
Photoluminescence and extinction spectra of silver nanoparticles in water. **a** The peak position of PL as the function of NP size. *Blue dots* correspond to PL emission peak; *red dots* correspond to optical density (extinction). **b** Extinction spectra. **c** Photoluminescence emission spectra of nanoparticles of different sizes

The raw synthetic product had a broad size distribution as was indicated by transmission electron microscopy (TEM) and extinction spectroscopy. In order to study the size dependence of the PL, this raw product was centrifuged to separate the particles by size. Relative centrifugal forces (RCF) of 500*g* to 14,000*g* were used. As a result, four samples were obtained with different size distributions (Fig. 1). The extinction spectra of the NPs separated by size are shown in Fig. 2b with the corresponding PL spectra displayed in Fig. 2c.

### TEM Size and Shape Characterization

Samples containing the desired NPs of different sizes were characterized by a JEOL JEM-100CX II transmission electron microscope. Images of selected samples and their size distributions are shown in Fig. 1. As was expected, the largest RCF applied resulted in the smallest NPs to be found in the supernatant (Fig. 1a), and the lowest RCF resulted in the largest NPs to be dragged into pellet (Fig. 1d).

### Quantum Yield

Relative QY was determined by comparing the photoluminescence from glycine-dimer-coated silver NPs to the well-characterized QY standard, quinine (in 0.05 M H_2_SO_4(aq)_). Each sample of NPs was matched in its optical density to that of the standard at the excitation wavelength of 366 nm (the QY of the quinine is known to be 0.54 ± 0.003 when excited at 366 nm) [21]. This condition guarantees that the NPs in the sample and the QY standard absorb the same amount of light upon excitation. Once the optical density of the standard and NP samples was matched, the PL spectra were recorded and the relative quantum yields of each sample were calculated using the ratio of the areas under their PL spectra.

### Bio-Imaging

A Leica TCS SP5 confocal microscope was used to obtain fluorescence and transmission images of neural and RBL cells incubated with NPs. The C17.2 cells were fixed using 1 % and RBL using 4 % paraformaldehyde for 20 min. The paraformaldehyde was then removed and cells were washed with PBS, covered with the media, and imaged with a ×40 oil objective. The excitation laser wavelength was 405 nm, and the emission channel was set to 420–535 nm. A 0.2-mL aqueous solution of fluorescent glycine-dimer-coated NPs with average diameter of 9 ± 4 nm was added to the fixed cells, which were contained in 2 mL of media. Imaging was performed every 15 min, starting immediately after addition of NPs. Every set of images included a z-stack of 100 focal planes. Images obtained of cells taken during NP incubation are shown on Figs. 6 and 7.

## Results and Discussion

### Intrinsic Silver NP Photoluminescence

After size separation, the samples contained NPs of mean silver core diameters 9 ± 4, 14 ± 5, 23 ± 4, and 32 ± 8 nm (Fig. 1). The smaller particles were near-spherical, while the larger particles were more irregular (Fig. 1a–d).

Optical properties of the particles were then studied to determine their size dependence. The intrinsic silver NP’s PL spectra were obtained with the excitation wavelength of 250 nm. This excitation wavelength is within the spectral region of the interband absorption for silver (the edge of the interband absorption is at 388 nm) [22]. Under a 250 nm excitation, the glycine dimer’s PL was not excited, and only the size-dependent intrinsic PL of the NPs was observed. It was found that as the NP’s diameter increased, the PL peak (Fig. 2c) was spectrally shifted towards the red end of the spectrum, similar to that of the SPR-related peak in absorbance, shown in Fig. 2b. This shift in absorbance is well-known and has been rationalized by the so-called *polaritonic* effect caused by a phase retardation of the electromagnetic waves (also known as *extrinsic* or *electrodynamic* effect) [13]. Multiple experimental studies confirm this effect and have shown a red shift of the SPR peak in the absorbance spectrum as the diameter of the NP increases (for diameters >10 nm) [23]; however, the same shift in the intrinsic PL has been previously reported in very few studies [11].

With the observed the shared trend in size dependence and spectral shifts of both the PL and absorbance (also called extinction or optical density (OD)) spectra (Fig. 2a) indicate that the intrinsic PL of the NPs is due a radiative decay of the SPR. The radiative decay of the SPR in noble metal NPs is also known as plasmon emission, or plasmon scattering, because of its very fast (a few femtoseconds) decay [12]. It has been stated that the red shift of both OD and PL spectra is an indicator of the plasmon emission, as opposed to the interband emission mechanism [11]. It is worth noting that some previous studies have observed PL due to both, the interband transitions and plasmon emission in noble metal NPs [11, 15, 24].

Comparison of the PL intensity from NPs of different sizes was performed for samples matched in OD at 250 nm. This guaranteed that the same number of photons was absorbed by all samples. The intensity of intrinsic PL from the silver NPs increased as their size increased. This is further evidence towards the rationalization of radiation damping as the source of PL in the metal NPs.

Briefly, the mechanism of PL due to a radiative decay is as follows: First, the interband electronic transitions are excited by the incident light. This excitation relaxes into the SPR modes, and the SPR then radiates or emits this energy as photons (radiatively decays) [11]. Seen as photoluminescence from the NPs, the energy radiated during this decay process is size-dependent because the SPR is also size-dependent. Evidence for such a PL mechanism in noble metal NPs has been shown in previous studies [24–26].

Despite the low QY observed from the intrinsic PL of metal NPs, much of the recent literature suggest its potential in bio-imaging and other applications [26]. An alternative use of SPR excitations in silver NPs can be found in the enhancement of PL from fluorescent ligands on the surface of a NP. In the next section, the size-dependent surface-enhanced PL of glycine dimer ligands is reported. Further discussion on how radiative decay affects the surface enhancement of the ligand PL is also provided.

### Surface-Enhanced Photoluminescence of Glycine Dimers

**Fig. 3:**
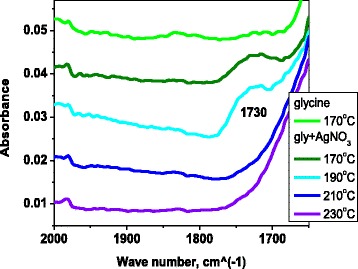
FTIR spectra of mixture of silver nitrate and glycine treated at different temperatures (*different colors of the curves*). The *upper curve* represents pure glycine without silver nitrate

During the NP synthesis, glycine forms fluorescent dimers, technically called pyrazine derivatives. The dimer formation has been previously reported under conditions different from those in our study and without the presence of silver nitrate or silver NPs [27, 28]. To our best knowledge, this study has been the first to demonstrate that fluorescent dimer formation is possible in the presence of silver and only takes place in a specific temperature range. This temperature range is the same range that produces the greatest yield of NP formation. The FTIR spectra illustrating this effect are shown in Fig. 3.

**Fig. 4:**
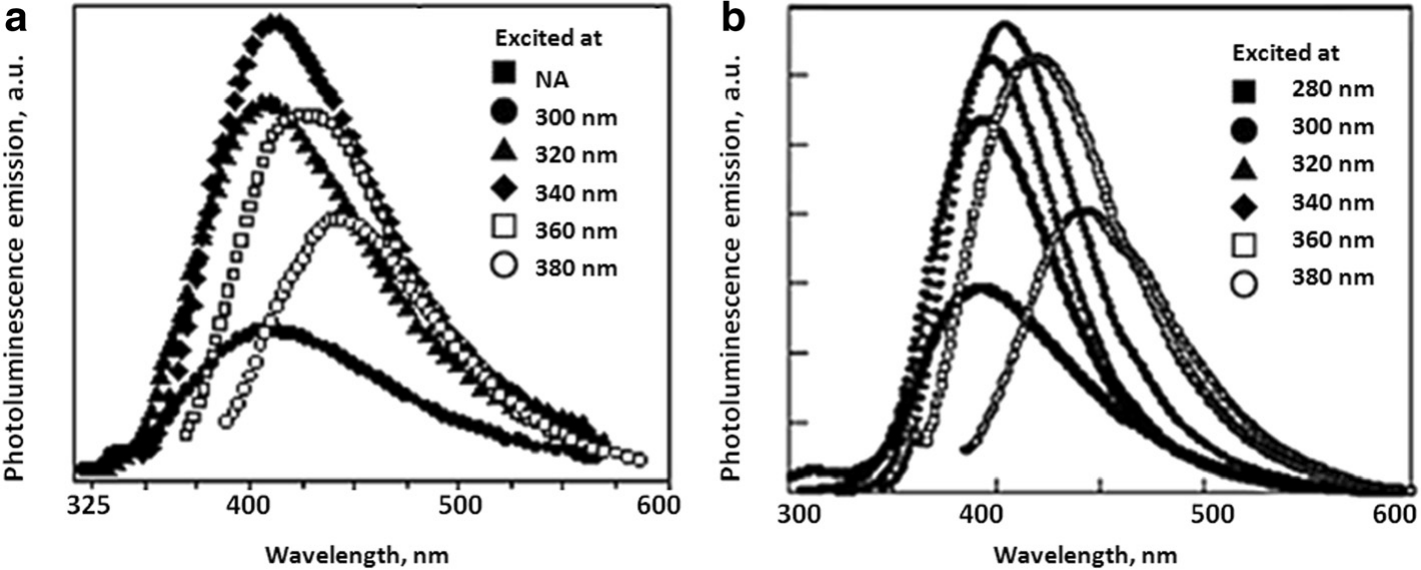
The excitation wavelength dependence of the photoluminescence emission spectra of **a** glycine-diemer-NPs of 9 nm average diameter and **b** free-standing glycine dimers [28]

In Fig. 3, the presence of glycine dimers was suggested by the peak around 1730 cm^−1^, which indicated peptide bond formation, as previously reported [27]. Further proof of the fluorescent glycine dimer formation in our experiments was the NP-glycine-dimer PL spectra (Fig. 4a). They were identical to the spectra of previously reported PL for the cyclic glycine dimers (Fig. 4b) [28]. The same dependence on the excitation wavelength (Fig. 4) as stated in the literature was also found.

Glycine was initially chosen as a NP-ligand because of the bio-compatibility of this amino acid. The bio-compatibility of the cyclic glycine dimers is yet to be studied.

**Fig. 5:**
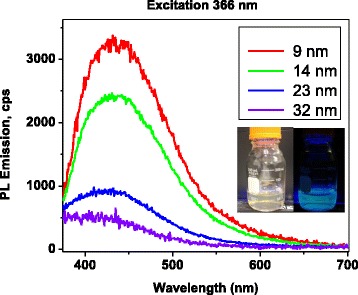
Photoluminescence emission spectra for NPs of different sizes when excited at 366 nm

It was found that the quantum yield for the glycine-dimer-NPs was non-monotonically dependent on the NP’s diameter, as it reached a maximum for the 23-nm-core-diameter NPs. Mainly, for the NP-ligand system, with a mean core diameter of 9 nm, the QY was 2.7 %; for 23-nm core, it was 5.2 %; and for 32 nm, it was 1.5 %. The dimer-NP emission spectral position, however, did not show any noticeable size dependence (Fig.5), which supports the hypothesis that the origin of this PL is glycine dimer’s emission.

The size dependence of the QY of a dimer-NP system was rationalized by the fact that local field on the NP’s surface is also size-dependent [10]. The local field is dampened via few mechanisms, including the radiative decay, discussed in a previous section. The radiative decay increases with the increase of NP size [23]. This leads to stronger damping of a local field for larger NPs. On the other end of the NP size range, as diameter of NP decreases further, the internal size effect dampens the local electric field at its surface [29]. Hence, there exists an optimal size of NPs, for which local field is maximum, explaining the experimental observations of maximum PL enhancement of the ligand’s emission by NPs of a specific diameter .

### Bio-Imaging

In order to evaluate the possible use of glycine-dimer-NPs as biomarkers, fixed neural stem and RBL cells were incubated with NPs. Fluorescence confocal microscopy was then used to study the particles’ penetration into the cells.

**Fig. 6:**
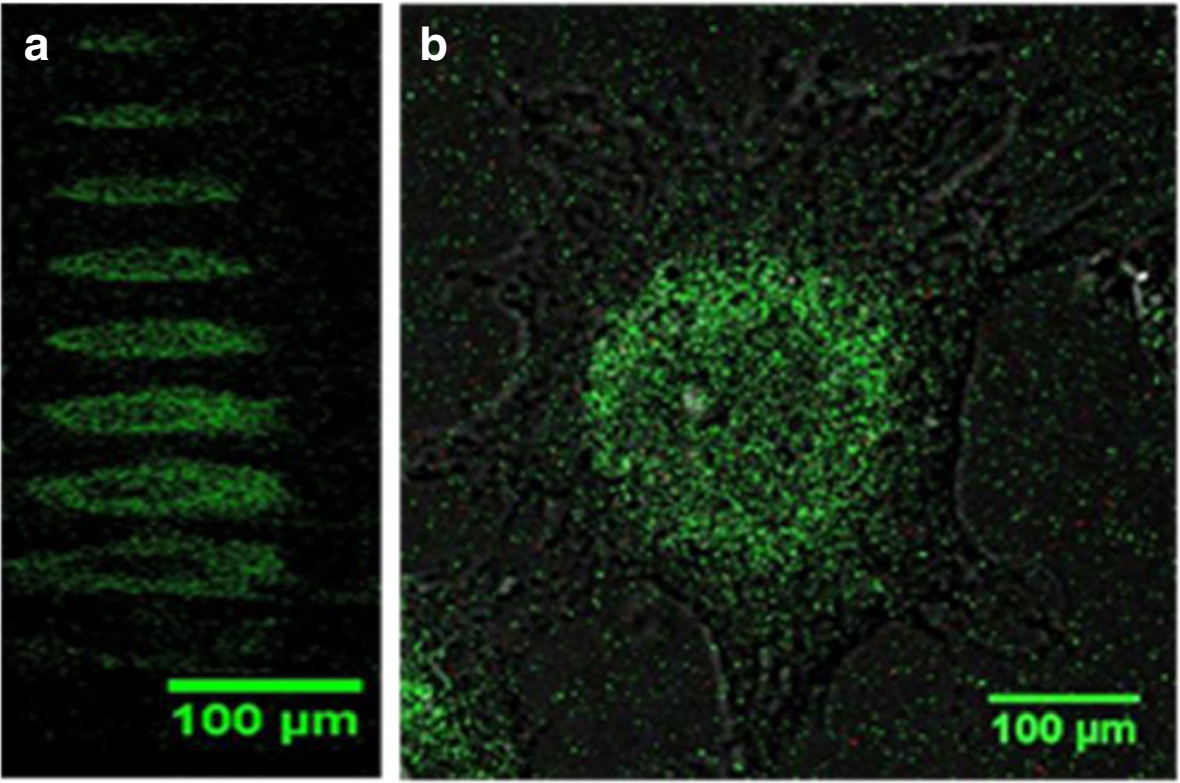
Fluorescent and transmission confocal microscopy image of a neural stem cell. **a** Cross-section (z-stacks) of a cell separated by 1 μm. **b** Top view of a cell as a sum of all z-stacks

**Fig. 7:**
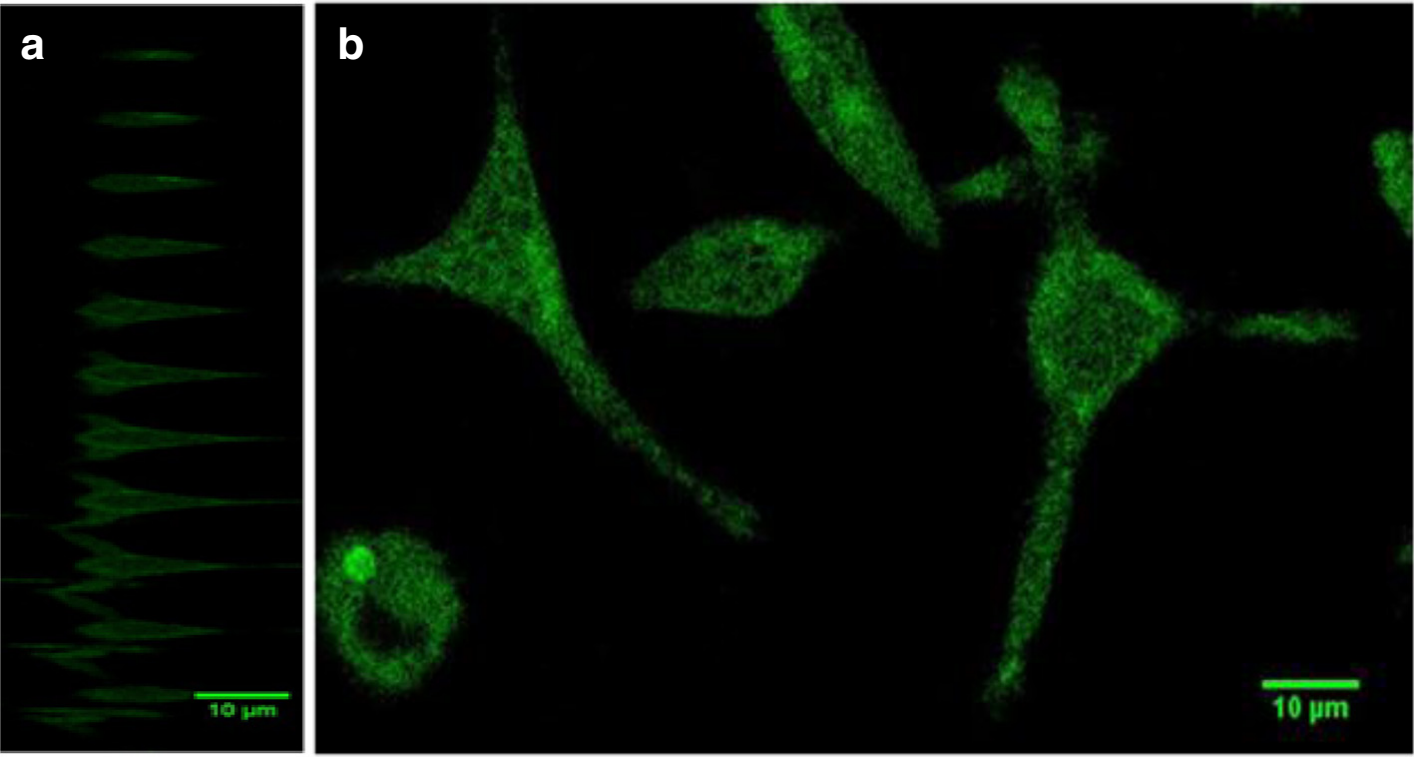
Fluorescent confocal microscopy image of the rat basophilic leukemia cells. **a** Cross-section (z-stacks) of a cell separated by 5 μm. **b** Top view of cells as at one specific z-stack

The smallest NPs (shown in Fig. 1a) were chosen as bio-imaging markers, since small NPs penetrate cell membranes more readily [30]. As one can see in Figs. 6 and 7, the majority of NPs accumulated on the fixed cells’ membrane, while fewer found in its body (dark area in the center). The fluorescence signal seen in the background of Fig. 6 was most likely caused by the presence of residual particles in the cell media. Images in Fig. 6 were obtained after 1 h 25 min of NP incubation.

In living cells, the membrane selectively controls transport of ions, large bio-molecules, and NPs into and out of the cell. The mechanisms of this transport depend on the size of the object being transported and include active endocytosis/exocytosis and passive transport (this mechanism is predominant in red blood cells). Internalization of a NP not only depends on their size, shape, surface charge, and surface chemistry [31] but also depends on the cell-specific parameters, such as cell type or cell cycle phase [32].

In this study, cells were fixed, then stained with NPs (as often performed in immunoassays), and hence only passive transport could take place. According to Van Lehn et al., pore formation in the membrane can be avoided if the ligand layer on the NP is able to adjust to the membrane, allowing surface charges to rearrange, so that the NP appears hydrophobic [33]. As the ligand layer around smaller particles contains a large amount of free volume because of the high curvature, ligand fluctuations are maximized, allowing small NPs to more easily penetrate a membrane [30]. We note that another possibility of NP internalization in this study is the partial permeabilization of a cell membrane by paraformaldehyde. Further study of the size-dependent NP internalization by live cells would be of great interest.

### Toxicity for the RBL Cells

**Fig. 8:**
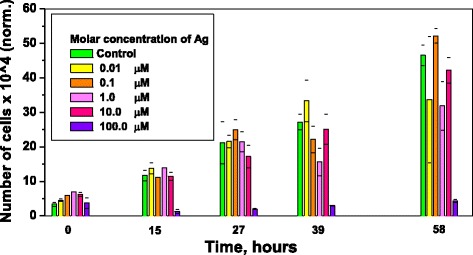
Proliferation of the RBL cells incubated with silver nanoparticles colloids of different concentrations

A total of 7 × 10^4^ RBL cells were cultured per well in a 24-well tissue culture plate. After a 17-h incubation period, aqueous suspensions of silver nanoparticles with specified NP concentrations (0.005, 0.05, 0.5, 5, and 50 nM) were added. These concentrations correspond to 0.01-, 0.1-, 1-, 10-, and 100-μM silver atom concentration, assuming one 10-nm NP contained 5 × 10^4^ silver atoms. Pure cells, without the addition of nanoparticles, were used as a control. The numbers of cells in each well were then counted at hour 15, 27, 39, and 58, after the silver nanoparticles were added. The data were acquired in triplicates. Figure 8 shows that NPs were non-toxic up to a 10-μM silver concentration and inhibited cell proliferation at a concentration of 100 μM.

Silver NPs are known to have antibacterial properties [34]. They are toxic towards bacterial cells but non-toxic towards mammalian cells up to relatively high concentrations [35]. This property of NPs is becoming more of interest, as antibiotic resistance in bacteria has become a major clinical and public health problem [36]. The main mechanism of NP toxicity is inhibition of the cell proliferation due to NP decomposition onto Ag^+^ ions. This decomposition is a result of dissolution of NPs in the liquid medium and digestion of NPs upon intercellular uptake. Ag^+^ ions can bind to thiol-containing molecules within the cell [37]. It has been shown that it leads to cell membrane detachment from the cell wall, cell wall damage, cell shrinkage and dehydration, and stress response via accumulation of DNA in the center of the cell [38]. In *Escherichia coli* bacteria, silver NPs cause cell proliferation inhibition [34]. Minimal inhibition concentration of Ag NPs has been determined to be 3.3 nM, which is relatively low compared to the Ag NPs concentration in our study (compare to 50 nM, for which the toxic effect was observed). Furthermore, other studies have observed that the toxicity of silver NPs versus Ag^+^ ions on mammalian cells showed significant toxic effects starting from 200 nM NP concentration [35], which suggests that our NPs are more toxic. Literature also suggests that other effects on the rat basophilic leukemia cells, such as degranulation, may occur [39]. We would like to add, that according to the experimental evidence presented in this work the origin of the photoluminescence of silver nanoclusters reported in previous publication [40] must be glycine dimers, and not the intrinsic emission of the silver clusters, as we suggested before.

## Conclusions

In conclusion, a correlation between the SPR-related peaks in the extinction and intrinsic emission spectra of silver nanoparticles of different sizes has been experimentally demonstrated. It was found that for NPs with an average diameter in the range of 9–32 nm, an increase in particle size leads to a red shift of the peak in both extinction and intrinsic photoluminescence spectra. These observations expand previous studies and prove the role of the SPR in the PL of noble metal NPs. The intrinsic particle PL emission was rationalized via radiative damping of the surface plasmon. In addition, the size-dependent quantum yield of the PL for the glycine dimer-coated NPs was studied. A quantum yield of the NP-glycine-dimer complex was shown to depend on silver particle's core size (maximum QY of (5.1 ± 0.1) % was estimated for averge NP's diameter 23+/-4 nm). Additionally, it was shown that NPs with average diameters of 9 ± 4 nm can be used for bio-imaging of fixed C17.2 neural stem cells as well as RBL cells. For RBL cells, nanoparticles were non-toxic up to a 10-μM silver concentration and inhibition of cell proliferation occurred at a concentration of 100 μM.
